# *N*′-[(*E*)-1-(5-Bromo-2-hy­droxy­phen­yl)ethyl­idene]-2-chloro­benzohydrazide

**DOI:** 10.1107/S1600536811048495

**Published:** 2011-11-19

**Authors:** Jian-Guo Chang

**Affiliations:** a Department of Materials Science and Chemical Engineering, Taishan University, 271021 Taian, Shandong, People’s Republic of China

## Abstract

The title compound, C_15_H_12_BrClN_2_O_2_, was synthesized by the condensation of 1-(5-bromo-2-hy­droxy­phen­yl)ethanone with 2-chloro­benzohydrazide in anhydrous ethanol. The Schiff base mol­ecule displays a *trans* configuration with respect to the C=N double bond. The dihedral angle between the two benzene rings is 13.74 (3)°. The mol­ecular conformation is stabilized by an intra­molecular O—H⋯N and the crystal structure by inter­molecular N—H⋯O hydrogen bonds.

## Related literature

For further details of the chemistry of the title compound, see: Carcelli *et al.* (1995 ▶); Salem (1998 ▶). For a related structure, see: Chang (2008 ▶).
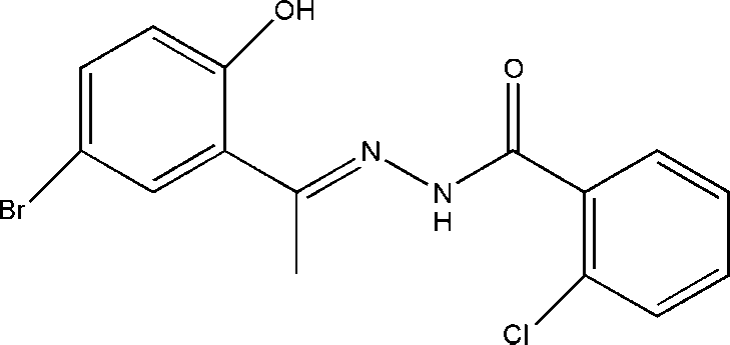


## Experimental

### 

#### Crystal data

C_15_H_12_BrClN_2_O_2_
*M* *_r_* = 367.62
Monoclinic, 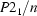

*a* = 14.861 (3) Å
*b* = 4.837 (1) Å
*c* = 21.310 (4) Å
β = 106.099 (4)°
*V* = 1471.7 (5) Å^3^
*Z* = 4
Mo *K*α radiation
μ = 2.98 mm^−1^
*T* = 298 K
0.15 × 0.10 × 0.06 mm

#### Data collection

Bruker APEXII CCD area-detector diffractometer
Absorption correction: multi-scan (*SADABS*; Sheldrick, 2003 ▶) *T*_min_ = 0.664, *T*_max_ = 0.842
7095 measured reflections
2605 independent reflections
1514 reflections with *I* > 2σ(*I*)
*R*_int_ = 0.052

#### Refinement

*R*[*F* ^2^ > 2σ(*F* ^2^)] = 0.043
*wR*(*F* ^2^) = 0.119
*S* = 1.05
2605 reflections
191 parameters
H-atom parameters constrained
Δρ_max_ = 0.31 e Å^−3^
Δρ_min_ = −0.42 e Å^−3^

### 

Data collection: *APEX2* (Bruker, 2005 ▶); cell refinement: *SAINT* (Bruker, 2005 ▶); data reduction: *SAINT*; program(s) used to solve structure: *SHELXS97* (Sheldrick, 2008 ▶); program(s) used to refine structure: *SHELXL97* (Sheldrick, 2008 ▶); molecular graphics: *XP* in *SHELXTL* (Sheldrick, 2008 ▶); software used to prepare material for publication: *SHELXTL*.

## Supplementary Material

Crystal structure: contains datablock(s) global, I. DOI: 10.1107/S1600536811048495/pk2360sup1.cif

Structure factors: contains datablock(s) I. DOI: 10.1107/S1600536811048495/pk2360Isup2.hkl

Supplementary material file. DOI: 10.1107/S1600536811048495/pk2360Isup3.cml

Additional supplementary materials:  crystallographic information; 3D view; checkCIF report

## Figures and Tables

**Table 1:** Hydrogen-bond geometry (Å, °)

*D*—H⋯*A*	*D*—H	H⋯*A*	*D*⋯*A*	*D*—H⋯*A*
O2—H2⋯N2	0.82	1.82	2.530 (4)	144
N1—H1⋯O1^i^	0.86	2.16	2.858 (4)	138

Symmetry code: (i) 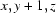.

**Fig. 1.**
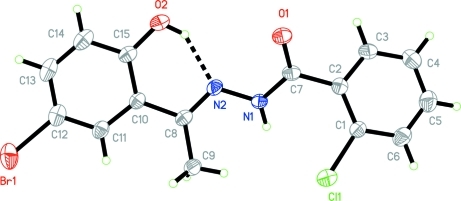
The molecular structure of compound (I). Displacement ellipsoids are drawn at the 30% probability level. Dashed lines show intramolecular O–H···N hydrogen bonds.

**Fig. 2.**
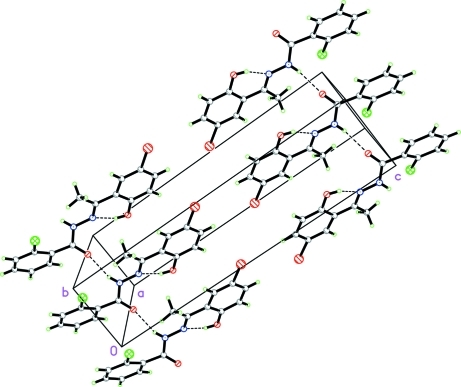
Packing diagram of (I), Showing intermolecular N—H···O hydrogen bonds as dashed lines.

## supplementary crystallographic information

### Comment

The chemistry of aroylhydrazones continues to attract much attention due to their ability to coordinate to metal ions (Salem, 1998) and their biological activity (Carcelli *et al.*, 1995).  As an extension of work on the structural characterization of aroylhydrazone derivatives (Chang, 2008), the title compound, (I), was synthesized and its crystal structure is reported here.

The title molecule displays a trans conformation with respect to the C8=N2 double bond (Fig. 1). The dihedral angle between the two benzene rings is 13.74 (3) °. The crystal structure is stabilized by an intramolecular O—H···N and by intermolecular N—H···O hydrogen bonds. (see Table 1 and Figs. 1 & 2.).

### Experimental

2-chlorobenzohydrazide (0.01 mol,1.71 g) was dissolved in anhydrous ethanol (40 ml), and 1-(5-bromo-2-hydroxyphenyl)ethanone (0.01 mol, 2.15 g) was added. The reaction mixture was refluxed for 4 h with stirring, then the resulting precipitate was collected by filtration, washed several times with ethanol and dried *in vacuo* (yield 85%). The compound (1.0 mmol, 0.36 g) was dissolved in dimethylformamide (10 ml) and kept at room temperature for 30 d to obtain colourless single crystals suitable for X-ray diffraction.

### Refinement

All H atoms were positioned geometrically and treated as riding on their parent atoms, with *C*—*H*(methyl) = 0.96 Å, C—H(aromatic) = 0.93 Å, O—H = 0.82 Å, *N*—H =0.86 Å and with *U*_iso_(H) = 1.5*U*_eq_(C_methyl_, O) and 1.2*U*_eq_(C_aromatic_, N).

### Crystal data

**Table:**

C_15_H_12_BrClN_2_O_2_	*F*(000) = 736
*M**_r_* = 367.62	*D*_x_ = 1.655 Mg m^−^^3^
Monoclinic, *P*2_1_/*n*	Mo *K*α radiation, λ = 0.71073 Å
Hall symbol: -P 2yn	Cell parameters from 1463 reflections
*a* = 14.861 (3) Å	θ = 2.9–22.6°
*b* = 4.837 (1) Å	µ = 2.98 mm^−^^1^
*c* = 21.310 (4) Å	*T* = 298 K
β = 106.099 (4)°	Block, colourless
*V* = 1471.7 (5) Å^3^	0.15 × 0.10 × 0.06 mm
*Z* = 4	

### Data collection

**Table:**

Bruker APEXII CCD area-detector diffractometer	2605 independent reflections
Radiation source: fine-focus sealed tube	1514 reflections with *I* > 2σ(*I*)
graphite	*R*_int_ = 0.052
φ and ω scans	θ_max_ = 25.1°, θ_min_ = 1.5°
Absorption correction: multi-scan (*SADABS*; Sheldrick, 2003)	*h* = −17→12
*T*_min_ = 0.664, *T*_max_ = 0.842	*k* = −5→5
7095 measured reflections	*l* = −25→25

### Refinement

**Table:**

Refinement on *F*^2^	Primary atom site location: structure-invariant direct methods
Least-squares matrix: full	Secondary atom site location: difference Fourier map
*R*[*F*^2^ > 2σ(*F*^2^)] = 0.043	Hydrogen site location: inferred from neighbouring sites
*wR*(*F*^2^) = 0.119	H-atom parameters constrained
*S* = 1.05	*w* = 1/[σ^2^(*F*_o_^2^) + (0.049*P*)^2^ + 0.1072*P*] where *P* = (*F*_o_^2^ + 2*F*_c_^2^)/3
2605 reflections	(Δ/σ)_max_ < 0.001
191 parameters	Δρ_max_ = 0.31 e Å^−^^3^
0 restraints	Δρ_min_ = −0.42 e Å^−^^3^

### Special details

**Table:**

Geometry. All e.s.d.'s (except the e.s.d. in the dihedral angle between two l.s. planes) are estimated using the full covariance matrix. The cell e.s.d.'s are taken into account individually in the estimation of e.s.d.'s in distances, angles and torsion angles; correlations between e.s.d.'s in cell parameters are only used when they are defined by crystal symmetry. An approximate (isotropic) treatment of cell e.s.d.'s is used for estimating e.s.d.'s involving l.s. planes.
Refinement. Refinement of *F*^2^ against ALL reflections. The weighted *R*-factor *wR* and goodness of fit *S* are based on *F*^2^, conventional *R*-factors *R* are based on *F*, with *F* set to zero for negative *F*^2^. The threshold expression of *F*^2^ > σ(*F*^2^) is used only for calculating *R*-factors(gt) *etc*. and is not relevant to the choice of reflections for refinement. *R*-factors based on *F*^2^ are statistically about twice as large as those based on *F*, and *R*- factors based on ALL data will be even larger.

### Fractional atomic coordinates and isotropic or equivalent isotropic displacement parameters (Å^2^)

**Table:**

	*x*	*y*	*z*	*U*_iso_*/*U*_eq_	
Br1	0.10714 (4)	0.26900 (11)	0.07214 (2)	0.0735 (3)	
Cl1	0.30550 (9)	0.2243 (3)	0.53343 (6)	0.0666 (4)	
O1	0.1050 (2)	−0.4214 (6)	0.45813 (14)	0.0539 (8)	
O2	−0.0140 (2)	−0.3493 (7)	0.27979 (16)	0.0590 (9)	
H2	0.0073	−0.2826	0.3163	0.089*	
N1	0.1335 (2)	0.0122 (7)	0.43053 (16)	0.0427 (9)	
H1	0.1565	0.1729	0.4427	0.051*	
N2	0.0972 (2)	−0.0529 (7)	0.36469 (16)	0.0422 (9)	
C1	0.2301 (3)	0.0827 (8)	0.5746 (2)	0.0408 (11)	
C2	0.1587 (3)	−0.0978 (8)	0.5449 (2)	0.0383 (10)	
C3	0.1083 (3)	−0.2171 (8)	0.5835 (2)	0.0501 (12)	
H3	0.0615	−0.3444	0.5651	0.060*	
C4	0.1260 (4)	−0.1510 (11)	0.6491 (3)	0.0591 (14)	
H4	0.0914	−0.2340	0.6743	0.071*	
C5	0.1939 (4)	0.0354 (11)	0.6766 (2)	0.0629 (14)	
H5	0.2046	0.0836	0.7203	0.076*	
C6	0.2466 (3)	0.1522 (10)	0.6402 (2)	0.0544 (13)	
H6	0.2935	0.2781	0.6593	0.065*	
C7	0.1312 (3)	−0.1848 (8)	0.4744 (2)	0.0398 (11)	
C8	0.1261 (3)	0.0878 (8)	0.3226 (2)	0.0379 (11)	
C9	0.1998 (3)	0.3071 (9)	0.3396 (2)	0.0505 (12)	
H9A	0.1715	0.4847	0.3275	0.076*	
H9B	0.2457	0.2737	0.3165	0.076*	
H9C	0.2294	0.3036	0.3858	0.076*	
C10	0.0828 (3)	0.0126 (8)	0.25362 (19)	0.0383 (10)	
C11	0.1075 (3)	0.1475 (9)	0.2027 (2)	0.0451 (11)	
H11	0.1523	0.2868	0.2132	0.054*	
C12	0.0688 (3)	0.0840 (10)	0.1384 (2)	0.0523 (13)	
C13	0.0016 (3)	−0.1209 (11)	0.1220 (2)	0.0579 (14)	
H13	−0.0257	−0.1645	0.0783	0.070*	
C14	−0.0251 (4)	−0.2602 (10)	0.1702 (3)	0.0629 (14)	
H14	−0.0704	−0.3978	0.1587	0.075*	
C15	0.0147 (3)	−0.1986 (9)	0.2358 (2)	0.0451 (12)	

### Atomic displacement parameters (Å^2^)

**Table:**

	*U*^11^	*U*^22^	*U*^33^	*U*^12^	*U*^13^	*U*^23^
Br1	0.1016 (5)	0.0720 (4)	0.0474 (3)	0.0180 (3)	0.0216 (3)	0.0081 (3)
Cl1	0.0595 (9)	0.0796 (10)	0.0582 (8)	−0.0267 (7)	0.0123 (6)	−0.0046 (7)
O1	0.081 (2)	0.0263 (18)	0.0544 (19)	−0.0110 (15)	0.0179 (16)	−0.0075 (15)
O2	0.070 (2)	0.048 (2)	0.059 (2)	−0.0214 (16)	0.0164 (19)	−0.0124 (18)
N1	0.061 (3)	0.0243 (19)	0.039 (2)	−0.0107 (17)	0.0094 (18)	−0.0010 (17)
N2	0.055 (3)	0.030 (2)	0.037 (2)	−0.0065 (17)	0.0073 (18)	−0.0052 (17)
C1	0.041 (3)	0.036 (2)	0.042 (3)	0.001 (2)	0.006 (2)	−0.001 (2)
C2	0.042 (3)	0.030 (2)	0.041 (2)	0.007 (2)	0.009 (2)	0.002 (2)
C3	0.058 (3)	0.036 (3)	0.056 (3)	−0.008 (2)	0.015 (3)	−0.002 (2)
C4	0.069 (4)	0.054 (3)	0.059 (3)	0.000 (3)	0.027 (3)	0.006 (3)
C5	0.073 (4)	0.066 (4)	0.049 (3)	0.003 (3)	0.017 (3)	−0.005 (3)
C6	0.057 (3)	0.047 (3)	0.054 (3)	−0.001 (2)	0.007 (3)	−0.009 (2)
C7	0.043 (3)	0.025 (3)	0.051 (3)	0.0008 (19)	0.013 (2)	−0.001 (2)
C8	0.038 (3)	0.028 (2)	0.045 (3)	0.0042 (19)	0.007 (2)	−0.006 (2)
C9	0.054 (3)	0.047 (3)	0.045 (3)	−0.011 (2)	0.005 (2)	0.001 (2)
C10	0.041 (3)	0.031 (2)	0.041 (3)	0.005 (2)	0.008 (2)	−0.005 (2)
C11	0.054 (3)	0.036 (3)	0.043 (3)	0.003 (2)	0.009 (2)	−0.007 (2)
C12	0.060 (3)	0.046 (3)	0.045 (3)	0.017 (3)	0.006 (2)	−0.002 (2)
C13	0.065 (4)	0.055 (3)	0.044 (3)	0.017 (3)	−0.002 (3)	−0.011 (3)
C14	0.060 (4)	0.061 (4)	0.056 (3)	−0.001 (3)	−0.003 (3)	−0.014 (3)
C15	0.040 (3)	0.042 (3)	0.049 (3)	−0.001 (2)	0.005 (2)	−0.004 (2)

### Geometric parameters (Å, °)

**Table:**

Br1—C12	1.889 (5)	C5—C6	1.368 (7)
Cl1—C1	1.743 (4)	C5—H5	0.9300
O1—C7	1.228 (5)	C6—H6	0.9300
O2—C15	1.346 (5)	C8—C10	1.477 (5)
O2—H2	0.8200	C8—C9	1.495 (6)
N1—C7	1.342 (5)	C9—H9A	0.9600
N1—N2	1.393 (4)	C9—H9B	0.9600
N1—H1	0.8600	C9—H9C	0.9600
N2—C8	1.291 (5)	C10—C11	1.399 (6)
C1—C2	1.384 (5)	C10—C15	1.413 (6)
C1—C6	1.392 (6)	C11—C12	1.366 (6)
C2—C3	1.383 (6)	C11—H11	0.9300
C2—C7	1.505 (6)	C12—C13	1.382 (7)
C3—C4	1.386 (6)	C13—C14	1.376 (7)
C3—H3	0.9300	C13—H13	0.9300
C4—C5	1.359 (7)	C14—C15	1.391 (7)
C4—H4	0.9300	C14—H14	0.9300
			
C15—O2—H2	109.5	N2—C8—C9	124.6 (4)
C7—N1—N2	117.6 (3)	C10—C8—C9	120.3 (4)
C7—N1—H1	121.2	C8—C9—H9A	109.5
N2—N1—H1	121.2	C8—C9—H9B	109.5
C8—N2—N1	118.1 (3)	H9A—C9—H9B	109.5
C2—C1—C6	120.6 (4)	C8—C9—H9C	109.5
C2—C1—Cl1	122.5 (3)	H9A—C9—H9C	109.5
C6—C1—Cl1	116.9 (3)	H9B—C9—H9C	109.5
C3—C2—C1	117.7 (4)	C11—C10—C15	116.8 (4)
C3—C2—C7	115.8 (4)	C11—C10—C8	121.4 (4)
C1—C2—C7	126.5 (4)	C15—C10—C8	121.8 (4)
C2—C3—C4	121.4 (4)	C12—C11—C10	123.0 (4)
C2—C3—H3	119.3	C12—C11—H11	118.5
C4—C3—H3	119.3	C10—C11—H11	118.5
C5—C4—C3	119.9 (5)	C11—C12—C13	119.3 (5)
C5—C4—H4	120.1	C11—C12—Br1	120.9 (4)
C3—C4—H4	120.1	C13—C12—Br1	119.8 (4)
C4—C5—C6	120.2 (5)	C14—C13—C12	119.9 (5)
C4—C5—H5	119.9	C14—C13—H13	120.0
C6—C5—H5	119.9	C12—C13—H13	120.0
C5—C6—C1	120.1 (5)	C13—C14—C15	121.1 (5)
C5—C6—H6	120.0	C13—C14—H14	119.5
C1—C6—H6	120.0	C15—C14—H14	119.5
O1—C7—N1	122.2 (4)	O2—C15—C14	117.1 (4)
O1—C7—C2	121.3 (4)	O2—C15—C10	123.1 (4)
N1—C7—C2	116.4 (3)	C14—C15—C10	119.8 (5)
N2—C8—C10	115.1 (4)		
			
C7—N1—N2—C8	−156.6 (4)	N1—N2—C8—C9	3.2 (6)
C6—C1—C2—C3	−3.5 (6)	N2—C8—C10—C11	−179.9 (4)
Cl1—C1—C2—C3	174.4 (3)	C9—C8—C10—C11	−1.5 (6)
C6—C1—C2—C7	178.1 (4)	N2—C8—C10—C15	−0.1 (6)
Cl1—C1—C2—C7	−4.0 (6)	C9—C8—C10—C15	178.3 (4)
C1—C2—C3—C4	2.4 (6)	C15—C10—C11—C12	0.2 (6)
C7—C2—C3—C4	−179.0 (4)	C8—C10—C11—C12	−179.9 (4)
C2—C3—C4—C5	0.2 (7)	C10—C11—C12—C13	0.5 (7)
C3—C4—C5—C6	−1.8 (8)	C10—C11—C12—Br1	−178.7 (3)
C4—C5—C6—C1	0.7 (7)	C11—C12—C13—C14	−0.6 (7)
C2—C1—C6—C5	2.0 (7)	Br1—C12—C13—C14	178.6 (4)
Cl1—C1—C6—C5	−175.9 (4)	C12—C13—C14—C15	0.0 (7)
N2—N1—C7—O1	5.5 (6)	C13—C14—C15—O2	−178.6 (4)
N2—N1—C7—C2	−172.0 (3)	C13—C14—C15—C10	0.8 (7)
C3—C2—C7—O1	−35.1 (6)	C11—C10—C15—O2	178.4 (4)
C1—C2—C7—O1	143.3 (4)	C8—C10—C15—O2	−1.4 (7)
C3—C2—C7—N1	142.5 (4)	C11—C10—C15—C14	−0.9 (6)
C1—C2—C7—N1	−39.1 (6)	C8—C10—C15—C14	179.2 (4)
N1—N2—C8—C10	−178.4 (3)		

### Hydrogen-bond geometry (Å, °)

**Table:**

*D*—H···*A*	*D*—H	H···*A*	*D*···*A*	*D*—H···*A*
O2—H2···N2	0.82	1.82	2.530 (4)	144.
N1—H1···O1^i^	0.86	2.16	2.858 (4)	138.

Symmetry codes: (i) *x*, *y*+1, *z*.
